# A Comprehensive Review of θ-Series Precipitates in Aluminum Alloys

**DOI:** 10.3390/ma18235406

**Published:** 2025-11-30

**Authors:** Bin Chen

**Affiliations:** School of Materials Science and Engineering, Shanghai Jiao Tong University, Shanghai 200240, China; steelboy@sjtu.edu.cn

**Keywords:** θ-series precipitates, aluminum alloys, precipitation, θ′ phase (Al_2_Cu), Guinier-Preston (GP) zones

## Abstract

This review systematically synthesizes the research progress on θ-series precipitates. It traces the historical evolution of θ-series precipitate research, from the accidental discovery of age hardening in Duralumin to the atomic-scale insights enabled by advanced electron microscopy and computational methods. The precipitation sequence (supersaturated solid solution → GP zones → θ″ → θ′ → θ), transformation mechanisms, and interfacial characteristics of θ′/Al are comprehensively analyzed, with special attention to ongoing controversies such as the structure of GP zones and the pathways of θ″ → θ′ transition. Furthermore, the review discusses how alloying elements regulate θ′ stability through interfacial segregation, vacancy interactions, and co-precipitation effects. Critical unresolved challenges are highlighted, including the kinetic limitations of θ′ coarsening and the need for mechanistic studies on multi-element microalloying. This synthesis aims to provide a foundation for future research toward designing high-performance age-hardenable aluminum alloys.

## 1. Introduction

Aluminum alloys are widely used in aerospace, automotive, and construction industries due to their low density, high strength-to-weight ratio, and good corrosion resistance. Among them, age-hardenable alloys (e.g., 2xxx, 6xxx, 7xxx series) derive their strength primarily from nanoscale precipitates formed during aging treatment. There are also a large number of literature reviews on the precipitates in these aluminum alloys [1]. θ-series precipitates, particularly the θ′ phase, rank among the most extensively studied strengthening phases in Al-Cu-based alloys, with a well-documented role in precipitation hardening [2,3].

Research on θ-series precipitates dates back to the early 20th century, with landmark discoveries such as age hardening in Duralumin by Alfred Wilm in 1906. Over the years, advancements in microscopy (e.g., high-resolution transmission electron microscopy, HR-TEM) and computational materials science have deepened our understanding of θ precipitate formation, structure, and evolution. However, despite these advancements, the field is still marred by controversies, such as the exact structure of Guinier-Preston (GP) zones and the mechanisms of phase transitions between θ″ (GP II), θ′, and θ phases.

This paper aims to systematically review the research history of θ-series precipitates in aluminum alloys, comprehensively sort out the relevant research context, and provide a detailed overview of the latest research progress in this field in recent years, with the hope of offering valuable references and insights for subsequent studies.

## 2. Fundamentals of θ-Series Precipitates

### 2.1. Precipitation Mechanism

The precipitation process in aluminum alloys is a complex yet crucial phenomenon that significantly impacts their properties. The heat treatment for age-hardenable aluminum alloys typically consists of three key stages. First, solution treatment is carried out at elevated temperatures (typically 450–550 °C) to maximize the dissolution of solute atoms in the aluminum matrix. Subsequently, rapid quenching to retain these solutes in a supersaturated solid solution (SSSS) by inhibiting their precipitation. In the final stage, aging (either natural or artificial), the SSSS decomposes into fine intermetallic precipitates through solute partitioning, which drives nucleation and growth.

The equilibrium phase diagram of aluminum alloys is fundamental to precipitation behavior, as it dictates the partial solid solubility between the aluminum matrix and alloying elements. This restricted miscibility serves as the thermodynamic driving force for precipitate formation during aging. Additionally, the maximum solubility of solute atoms in the parent matrix should be sufficient to ensure adequate supersaturation upon quenching for subsequent precipitation reactions. Moreover, the solubility of solute atoms in aluminum must exhibit a strong temperature dependence with a negative gradient. That is, as the temperature decreases from the solution treatment range to the aging temperature, the solubility limit should drop significantly. This sharp reduction in solubility creates the necessary supersaturation condition for the nucleation and growth of coherent precipitates during thermal aging. Common alloying elements in aluminum matrices that facilitate precipitation include Cu, Mg, Si, Zn, and Li, which form coherent strengthening phases. For instance, in Al-Cu alloys, precipitation is thermodynamically driven by the negative solubility gradient of Cu in Al (decreasing solubility with lower temperature), the supersaturation of Cu in the SSSS after quenching, and the system’s tendency to minimize energy through phase separation and the formation of coherent precipitates.

The thermodynamic driving force for precipitation is fundamentally governed by the temperature-dependent solubility limits. While the equilibrium phase diagram delineates the solvus between the α-Al matrix and the stable θ-Al_2_Cu phase, the practical precipitation sequence involves metastable phases (GP zones, θ″, θ′) that possess their own distinct solvus lines. These metastable solvus lines, which lie within the equilibrium two-phase field, define the temperature-composition regions within which each precursor phase is stable or can form. The classic diagram effectively summarizes these relationships [4]. It illustrates, for instance, that GP zones are only stable at relatively low temperatures, above which they dissolve. The θ″ and θ′ phases are stable at progressively higher temperatures.

### 2.2. Definition and Structural Characteristics

The precipitation sequence in Al-Cu alloys follows a well-defined pathway. In the 1950s, using X-ray techniques, the generally accepted precipitation sequence was established [5,6,7]:

SSSS **→** GP zones (GP I) **→** θ” (GP II) (Al_3_Cu) **→** θ′ (Al_2_Cu) **→** θ (Al_2_Cu).

Initiating with the formation of GP zones during room temperature aging, these zones are composed of face-centered cubic copper clusters that maintain perfect coherency with the aluminum matrix. GP zones (GP I) are thin, disc-shaped clusters of Cu atoms (1–3 atomic layers) coherent with the Al matrix ({100} planes), formed at room temperature or low aging temperatures. It should be noted that the GP zones were often referred to as GP I zones to distinguish from θ”, which is also referred to as GP II. As the aging process progresses, these GP zones evolve into the θ” phase (Al_3_Cu), which has a disc-shaped morphology approximately 2 nm thick and 10 nm in diameter and exhibits a 2% lattice mismatch along the (001)_Al_ plane, creating significant coherency strains that contribute to strengthening, as described in. θ” (GP II) is a metastable phase with a tetragonal structure, maintaining coherency with the matrix and contributing significantly to strength. The θ” symbol is preferred by workers who consider that it is a coherent intermediate precipitate rather than a zone. The structure is tetragonal with a = b = 0.404 nm, and c = 0.76–0.80 nm being 0.8 nm when initially formed and changing to 0.76 nm as the zones grew. Further aging gives rise to the θ′ phase (Al_2_Cu) with a distinct body-centered tetragonal (BCT) structure (space group I4/mmm, a = 0.404 nm, c = 0.580 nm) [8], which forms on {100}_Al_ habit planes and acts as the primary strengthening phase. The orientation relationship between the θ′ phase and the Al matrix is (001)_θ′_//(001)_Al_ and [100]_θ′_//[100]_Al_ [9]. GP zones, θ”, and θ′ all have a disc-like habit and are parallel to {001} planes of the aluminum matrix. This semi-coherent intermediate phase ultimately transforms into the equilibrium θ phase (Al_2_Cu), which has a BCT structure (space group I4/mmm) and lattice parameters: a = 0.607 nm, c = 0.487 nm [10], but loses coherency with the matrix, resulting in reduced strengthening effects.

Table 1 summarizes the key crystallographic parameters, clearly illustrating the evolution in crystal structure, lattice parameters, and the loss of coherency from GP zones to the equilibrium θ phase [11].

Understanding the structural evolution of θ-series precipitates provides a foundation for examining their strengthening mechanisms, which are critical to the mechanical performance of age-hardenable aluminum alloys.

### 2.3. Precipitation Kinetics

The precipitation sequence of θ-series phases in Al-Cu alloys follows well-defined kinetic laws, with nucleation, growth, and coarsening behaviors quantified by classic models and experimental parameters from literature. Most models for precipitation kinetics in aluminium alloys follow a well-established approach, which combines the Johnson–Mehl–Avrami–Kolmogorov (JMAK) model for nucleation and growth with the Lifshitz–Slyozov–Wagner (LSW) theory for coarsening. The coupled JMAK and LSW approach offers a mathematically simpler alternative for studying precipitate nucleation, growth, and coarsening. This hybrid approach facilitates direct correlation with experimental data, thus enabling the efficient optimization of microstructures in novel multi-component materials.

The JMAK model was originally developed by independently by C. Kolmogorov [12], W. A. Johnson and R. F. Mehl [13], and M. Avrami [14,15,16], to describe recrystallization, was later successfully extended to model diffusion-controlled precipitation reactions [17,18,19,20,21]. In general, the transformation kinetics follow the Avrami equation [14,15,16,22]:

(1) $$f(t)=1-exp\left(-kt^{n}\right)$$

where *f* is the volume fraction of phases, *t* is aging time, *n* is the Avrami exponent reflecting growth geometry, and *k* is the temperature-dependent reaction rate constant which is generally expressed by the Arrhenius-type equation [23]:

(2) $$k=k_{0}exp\left(-\frac{E}{RT}\right)$$

where *k*_0_ is the frequency factor, *E* is the activation energy required for the diffusion of solute atoms, *R* is the ideal gas constant and *T* is the isothermal temperature in kelvin.

These kinetic parameters, activation energy *E* and Avrami parameter *n*, are most commonly derived from the analysis of isothermal differential scanning calorimetry (DSC) data [24,25,26,27], which serves as a primary experimental source for JMAK model fitting.

J. Karov and W. V. Youdelis [28] summarized prior research by observing that, contrary to the constant-growth assumption proposed by C. Wert and C. Zener [17,18,19], who assigned Avrami exponents *n* of 3/2, 5/2, and 2 for spheres, discs, and rods, respectively, F. S. Ham [20,21] demonstrated that growth conditions vary with supersaturation, leading to changes in both particle morphology and the growth constant. In F. S. Ham’s model, solute depletion is described by first-order kinetics that depend on the instantaneous size and shape of the particles. For instance, discs of constant eccentricity initially exhibit an *n*-value of 3/2, which shifts toward unity as inter-particle interactions alter their dimensions [21]. Constant-thickness discs begin with *n* = 2, while rods that grow primarily at their ends show *n* = 1. This kinetic framework is consistent with the aging behavior of the θ-series precipitates. In their own study on the θ′ phase, J. Karov and W. V. Youdelis [28] found that the precipitation of the θ′ phase follows Avrami kinetics, with a growth constant of *n* = 1.68. In addition, the precipitation of *θ* also conforms to Avrami kinetics, characterized by an Avrami exponent of *n* = 0.63. These parameters support a growth model in which the equilibrium θ phase forms via the dissolution of θ′ particles, with solute being supplied to the growing θ phase through dislocation pipelines from the concurrently dissolving θ′ particles. Higher Avrami exponents have been reported in other studies, with values of *n* = 1.9 for θ′ [24], 1.65 for θ [24], and 1.44 for the θ′/θ [25], respectively. However, lower Avrami exponents have also been reported in other studies, with values of *n* = 1.1 for θ′ [29]. The significant variation in reported Avrami exponents for the θ′ phase underscores the sensitivity of precipitation kinetics to experimental conditions.

Obtaining accurate Avrami exponent *n* values for the precipitation of GP zones and θ″ phases in Al-Cu alloys proves particularly challenging [29]. The primary difficulty arises from the overlapping nature of their transformation signals during DSC analysis. At extended aging times, θ″ coarsening adheres to the modified Lifshitz-Slyozov-Wagner (LSW) theory, originally proposed by I.M. Lifshitz and V.V.J. Slyozov [30] and C. Wagner [31], adapted for plate-like precipitates:

(3) $$D_{t}^{3}-D_{0}^{3}=K\cdot t$$

where *D_t_* is the average particle diameter at time *t*, *D*_0_ is the initial particle diameter, and *K* is the coarsening rate constant.

The kinetic equation modified by Boyd et al. [32] for disc-shaped particles in Al–Cu alloys, is expressed as follows:

(4) $$d_{2}^{3}-d_{1}^{3}=K\cdot \left(t_{2}-t_{1}\right)$$

where *d*_1_ and *d*_2_ are the average precipitate diameter at the times *t*_1_ and *t*_2_, respectively. Equation (4) can be applied to the coarsening of precipitates via solute diffusion through the matrix in case of plate-like particles [33].

In the study of J.D. Boyd and R.B. Nicholson [32], the measured coarsening kinetics of θ″ precipitates show good quantitative agreement with the LSW theory. In contrast, θ′ precipitates do not follow the simple LSW coarsening model, exhibiting coarsening rates considerably higher than predicted. This behavior is attributed to the combined effect of semi-coherent interfaces and the narrow inter-particle spacing, which facilitate short-circuit diffusion and particle coalescence. However, Z. Chen et al. [33] demonstrated that the coarsening behavior of θ′ precipitates in aged Al–Cu alloys at a certain ageing temperature (230, 260, 290, and 320 °C) follows the classical Lifshitz–Slyozov–Wagner (LSW) theory. P. Merle and F. Fouquet [34] further clarified that θ′ plates only approach LSW kinetics once their aspect ratio drops to ~20, while the time exponent remains below t^1^/^3^, indicating only partial compliance.

For the equilibrium θ phase, Brown [35] observed classic LSW behavior, confirming that fully incoherent, equiaxed θ particles strictly obey LSW ripening.

In addition to calorimetric techniques, the measurement of physical properties, particularly electrical resistivity/conductivity, has been a cornerstone in probing the very early stages of precipitation, where the formation of solute clusters and GP zones occurs [36,37,38]. These methods are exceptionally sensitive to the scattering of conduction electrons by point defects (vacancies) and solute atoms. During the decomposition of the SSSS, the reduction in solute concentration in the matrix and the formation of coherent precipitates lead to a characteristic evolution of electrical resistivity.

### 2.4. Precipitation Hardening Mechanism

The mechanical properties of certain metallic alloys, including age-hardenable aluminum alloys, can be substantially enhanced through the controlled formation of nanoscale, uniformly distributed secondary phase particles within the host lattice. The size, distribution, and coherency of precipitates exert a profound influence on the mechanical properties of aluminum alloys. For example, it has been found that approximately 10^16^ of these precipitates can be present in each cm^3^ of an Al-Cu-Mg-Ag alloy [1]. Microstructural stability is achieved when the hardening precipitates maintain crystallographic coherence and planar alignment with the parent matrix. Precipitates can exhibit a variety of morphologies, including spheres, cubes, discs, and needles. Their shape is determined by the misfit between the precipitates and the matrix, as well as interfacial energy [39]. In general, precipitates adopt a shape that minimizes the total energy associated with their formation. For instance, when the atomic misfit is very small, spherical particles form because a sphere has the lowest surface-to-volume ratio among geometric shapes. In contrast, when the atomic misfit is significant, particles typically take on a disc-like or needle-like shape to reduce strain energy. Cubes represent an intermediate morphology. As particles grow, coherent strains accumulate; these strains are eventually released when the particles lose coherency during the transition from the metastable phase to the equilibrium phase.

The primary strengthening mechanism in precipitation-hardened aluminum alloys is related to the interaction between these precipitates and dislocations. Depending on their size, distribution, and coherence with the matrix, precipitates can impede dislocation motion through mechanisms such as Orowan looping, direct cutting, or dislocation pile-up at precipitate-matrix interfaces. These interactions hinder dislocation glide and increase yield strength. When precipitates are non-shearable, strengthening is mainly controlled by the Orowan mechanism [40]. When dislocations encounter such precipitates, the dislocation line begins to bow between adjacent particles. As this bowing progresses, the dislocation segments reconnect at a critical curvature angle, ultimately forming loops that encircle the precipitates. The energy stored in these dislocation loops contributes to the alloy’s strengthening. Complementing this, the Frank-Read source mechanism also contributes to hardening [41]. Coherent precipitates, along with their associated lattice strain fields, can induce dislocation multiplication: the strain fields first cause localized dislocation bending; as stress increases, the dislocations develop multi-inflection curvature; and at critical stress levels, opposing dislocation segments annihilate, leaving residual loops around the precipitates [1].

GP I zones, θ” precipitates, and θ′ (Al_2_Cu) precipitates interact with dislocations in Al-Cu alloys distinctly based on their structure and size. According to the earlier understanding, while all these plate-shaped phases grow on {001}Al habit planes, dislocations would shear the fully coherent GP I zones and θ″ precipitates [42] but bypass the tetragonal θ′ (Al_2_Cu) precipitates [43]. Recent work by J. Yang et al. [44] has provided further insight: GP I zones (~10 nm) are easily sheared, preserving ductility with moderate strengthening. θ″ precipitates (5–12 nm) also undergo shear, though at higher stress, resulting in limited morphological change and balanced strength–ductility. θ′ precipitates exhibit size-dependent behavior: those below ~40 nm are transitionally shearable, whereas larger ones (>50 nm) are bypassed via Orowan looping.

Quantitatively, the strengthening contributions from different precipitates can be described using specific equations that account for their distinct interaction mechanisms with dislocations. For GP zones and θ” precipitates, the fully coherent nature and small size lead to modulus strengthening, where the mismatch in shear modulus between the zones and the Al matrix induces lattice strain to impede dislocation motion. This strengthening effect (critical resolved shear stress, CRSS) is described by the model proposed by Yan et al. [45]:

(5) $$∆\tau_{GP}=\frac{∆\mu }{4\pi \sqrt{2}}f_{GP}^{0.5}$$

where ∆μ is the difference in shear modulus between GP zones and the Al matrix, and $f_{GP}$ is the volume fraction of GP zones (ranging 0.01–0.06 in underaged conditions). This equation reflects the direct dependence of GP zone strengthening on their volume fraction, as their small size and coherence limit significant size-related variations in strain fields.

In contrast, θ′ precipitates are primarily bypassed by dislocations via the Orowan looping mechanism, as their size exceeds the critical shearable limit. For these plate-like precipitates (thickness w << diameter d), the CRSS contribution is described by the micromechanical model developed by Weakley-Bollin et al. [46] for θ′ in Al-Si-Cu alloys:

(6) $$\tau_{\theta^{’}}=0.13\left(\frac{Gb}{\sqrt{dw}}\right)\left(\sqrt{f}+0.75\sqrt{\frac{d}{w}}f+0.14\frac{d}{w}f^{3/2}\right)\left(\ln\frac{0.87\sqrt{dw}}{r_{0}}\right)$$

where *d*, *w*, and *f* are the precipitate diameter, thickness, and volume fraction of the precipitate, respectively, as a function of time; *G* is the Al matrix shear modulus; *b* is the Burgers vector; and *r_o_* is the inner cut-off radius, which is set equal to *b*. This equation emphasizes the critical role of inter-precipitate spacing in Orowan strengthening—smaller spacing increases the resistance to dislocation looping, thereby enhancing CRSS.

## 3. Historical Development of θ-Series Precipitate Research

### 3.1. Early Discoveries

Unlike bronze (dating back to at least 2500 B.C.) and iron (emerging around 1500 B.C.), aluminum was discovered and utilized much later. In 1800, Alessandro Volta developed the battery, and in 1807, British scientist Humphry Davy capitalized on this invention to isolate new metals from their salts through electrolysis, such as potassium from potash and sodium from soda. However, pure metallic aluminum had a major drawback for non-decorative applications as it was extremely soft and ductile. Similarly to how adding tin to copper created the harder alloy bronze and adding carbon to iron, with proper heat treatment, produced steel, efforts were made to enhance aluminum through appropriate treatments.

In 1906, a significant milestone in aluminum alloy development was reached when Alfred Wilm accidentally created the first age-hardenable aluminum alloy, known as Duralumin, during his experiments with an Al-Cu-Mn-Mg alloy [47,48]. After quenching, this alloy exhibited unexpected hardening without losing its ductility, while retaining its lightweight characteristics. However, a recent report [49] has revealed that even prior to Wilm’s breakthrough, the Wright brothers unknowingly benefited from age hardening during their historic first flight in 1903. The engine components of their aircraft were fabricated from an Al-8% Cu alloy that, though likely by chance, had undergone age-hardening.

The first detailed study was conducted by P. D. Merica et al. in 1919 [50]. They suggested that since copper’s solid solubility in aluminum decreases significantly with decreasing temperature, slowly cooling a 4% copper alloy would cause CuAl_2_ to precipitate from the initially homogeneous solid solution formed at the solution-treatment temperature. This implies a room-temperature microstructure with CuAl_2_ particles dispersed in the matrix, where the matrix’s copper concentration matches equilibrium diagram predictions. Walter Rosenhain was the first to put forward the idea that the maximum hardening effect in Al-Cu alloys is not necessarily linked to the existence of equilibrium phase particles [51]. His reasoning was that the formation of separate particles of this phase would lower the solute concentration in the solid solution, which should actually lead to softening instead of hardening. Instead, he proposed that age hardening arises from a higher degree of disorder in the parent lattice during the early stages of aging. Concurrently, Zay Jeffries and Robert Archer [52] proposed that the fine dispersion of CuAl_2_ in duralumin increases the hardness of pure aluminum by approximately 10 times, attributing this to strengthened lattice cohesion rather than the particles’ inherent hardness. They postulated a ‘keying action’ where CuAl_2_ particles hinder slip plane movement, with the mechanism depending on particle density: smaller, more numerous particles enhance strength by impeding deformation, while larger particles are less effective. This explains why aging initially hardens the alloy but reduces hardness once particles exceed a critical size and coalesce. In 1929, Marion L. V. Gayler and George D. Preston identified a two-stage process: Stage I (atomic segregation → increased hardness/resistance) and Stage II (coagulation → softening) [53]. In 1932, P. D. Merica [54] substantially revised his earlier theory by leveraging X-ray evidence of atomic diffusion from Hengstenberg and Wassermann [55], proposing that age hardening stems from aggregates or “knots” of Cu atoms, which are essential for forming the equilibrium precipitate, that distort the lattice before visible precipitation.

### 3.2. θ′ Precipitates

In 1932, F. Mehl et al. [56] published findings on the nature of precipitates in aluminum alloys containing copper and magnesium silicide, respectively. They characterized the precipitates in Al-Cu alloys, though they did not recognize that the phase they described would later be identified as θ′. They noted that the small size of the precipitates made their etching behavior too indistinct for reliable phase identification. However, based on the alignment of diffraction lines with those of CuAl_2_, which was consistent with Schmid and Wassermann’s earlier findings [57] of CuAl_2_ in over-aged 4–5% Cu alloys, they tentatively assigned the observed precipitates to CuAl_2_. They in the first case observed that the precipitation of CuAl_2_ from the solid solution occurred as plate-like structures, which were oriented parallel to the (100) planes of the solid solution lattice.

Critical evidence emerged in 1935 when Gustav Wassermann and Jakob Weerts [58] discovered the metastable θ′ phase in Al-Cu alloys via X-ray diffraction. θ′ has the same composition as CuAl_2_ but distinct lattice parameters, proving that aging involves multiple transitional phases. William L. Fink and Donald W. Smith [59,60] used metallography to observe precipitation on {111} slip planes before measurable hardening, challenging the knot theory.

While these early studies laid the groundwork, it was not until the late 1930s that more precise structural characterizations began to clarify the nature of θ′ precipitates. The pivotal work of G. D. Preston [61] provided the first definitive structural characterization of θ′, identifying its tetragonal structure and coherent interface with the Al matrix. This was quickly followed by the efforts of R. F. Mehl and L. K. Jetter [62], who integrated disparate theories to propose the now-classic continuous sequence of GP zones → θ′ → θ. Concurrently, M.L.V. Gayler [63,64] provided metallographic evidence for distinct microstructural stages during aging, further solidifying this conceptual framework. The collective impact of these studies was to resolve the earlier ‘pre-precipitation vs. precipitation’ debate, establishing that age hardening involved a series of well-defined metastable phases.

Prior studies, relying on X-ray diffraction alone, proposed the existence of an intermediate precipitate but could not visually characterize it. In 1939, a paper by Marie Gayler [65] provided a seminal microscopic analysis of intermediate phases in age-hardening Al-Cu alloys. Gayler’s key contribution was using metallographic microscopy to directly observe and distinguish between the metastable intermediate phase (termed α-CuAl_2_), which appears blue-grey and integrates with the aluminum matrix, and the stable β-CuAl_2_ phase. She concluded the transformation from α to β is polymorphic rather than a separate precipitation reaction.

Guinier’s 1942 study [66] provided groundbreaking insights into the θ′ phase in Al-Cu alloys, revealing it as a coherently precipitated metastable phase (Al_2_Cu, tetragonal a = 5.70 Å, c = 5.82 Å) that forms directly from pre-existing solute-enriched ordered zones within the aluminum matrix. Critically, he identified significant elastic strain interaction between the θ′ lattice and the matrix, evidenced by unique satellite diffraction spots corresponding to a ~10.1 Å periodicity, which stabilizes the coherent θ′ configuration.

In 1956, J. M. Silcock and T. J. Heal [43] revealed that the θ′ precipitate phase in Al-Cu alloys deviates significantly from the established distorted CaF_2_-type structure (Preston model). Through detailed X-ray intensity analysis, the authors demonstrate that neither simple composition changes nor positional distortions adequately explain the anomalously high intensities observed for specific reflection types. This combined modification (removing ~1/8 Al from Al sites and placing ~1/4 of those Al atoms into the “unoccupied” sites within the Cu layers) provides the best fit to the experimental intensity data. It successfully explains the enhanced intensity of reflections while maintaining random disorder to avoid introducing new diffraction spots. This results in a structure slightly richer in aluminum than the ideal CuAl_2_ stoichiometry.

In 1983, U. Dahmen and K. H. Westmacott [67] extended a precipitate plate growth model developed for Pt-C interstitial alloys to substitutional systems, comparing it with θ′ precipitation in Al-4% Cu. They suggested that θ′ forms from the solid solution through ledge growth on {001} planes, involving both conservative and non-conservative partial dislocations. Contrary to existing Al-Cu models assuming a/n [001] dislocations at ledges, single ledges (one θ′ unit cell high) are linked to partial dislocations with ½ [100] or ½ [010] shear components, analogous to 1/6 <112> Shockley partials in γ′ nucleation on {111} planes in Al-Ag, resulting from f.c.c. matrix-b.c.t. precipitate lattice correspondence. Double ledges (1.16 nm high) form with canceling shears to minimize shape change, while frequently observed superledges (2.03 nm high) further reduce volume change. Misfit accommodation on precipitate broad faces arises from epitaxial dislocations via growth partial combinations. The transformation from θ″ to θ′ is initiated by vacancy supersaturation from dissolving dislocation loops. The model’s validity is supported by common behaviors in Pt-C, Al-Cu, and Al-Ag systems, suggesting broad applicability.

Regarding the θ′ → θ transformation, two hypotheses have been proposed: one suggests that θ nucleates independently of θ′, with eventual θ′ dissolution occurring via counter-current diffusion through the alpha matrix; the other proposes that θ′ transforms directly into θ through a polymorphic process. In 1942, Guinier [66] demonstrated that the metastable θ′ phase transform to the stable θ phase occurs discontinuously via nucleation and growth at specific sites within θ′ (like Cu-rich planes or characteristic atomic clusters), resulting in multiple θ orientation variants, and crucially established that the final θ precipitate texture depends on thermal history, as slow cooling bypassing θ′ formation yields only a subset of orientations compared to aging through θ′. M. Von Heimendahl and G. Wasserman [68] concluded that θ forming within the matrix nucleates in dissolving θ′ plates and grows at the expense of the θ′ phase. These observations were later confirmed by Vaughan and Silcock [69] in a TEM study of bulk-aged Al-4% Cu alloy. C. Laird and H.I. Aaronson [70] demonstrated that three concurrent reactions occur during θ crystal formation: after θ nucleates at matrix/θ′ boundaries, it consumes the θ′ plates where it nucleated, the surrounding matrix transforms to θ simultaneously, and nearby θ′ plates dissolve as θ grows.

### 3.3. GP Zones (GP I Zones) and θ″ Precipitates (GP II Zones)

In contrast to the relatively well-defined θ′ phase, the study of GP zones (GP I zones) and θ″ precipitates (GP II zones) present greater complexity due to their nanoscale dimensions and the historical limitations of characterization techniques. Research on GP zones and θ″ precipitates present greater complexity compared to θ′ precipitates, primarily owing to their considerably reduced dimensions. Consequently, the two states were indistinguishable in the earliest studies, and the term “GP zones” used at that time implicitly covered both GP I zones and GP II zones.

The initial discovery of GP zones was independently discovered in the late 1930s in Al-Cu alloys by A. Guinier [71,72,73,74] and G. Preston [75], who used different X-ray techniques. Both Preston and Guinier observed streaks of varying lengths in their Laue photographs, which they attributed to small zones (presumably clusters of copper atoms) oriented along the {100} planes of aluminum. In their initial models, Guinier and Preston characterized GP zones as thin, Cu-enriched platelets that maintain coherence with the {100} planes of the aluminum matrix. Given that Cu atoms have a notably smaller atomic radius compared to Al (r_Al_ = 0.143 nm, r_Cu_ = 0.128 nm), the Al planes adjacent to the GP zones are positioned marginally closer to the Cu-rich layers. Several models for GP zones have been proposed. Building on his initial work, A. Guinier [76] further explored his experimental results, considering two hypotheses: atom segregation into matrix-structured “zones” and precipitation of small inclusions with progressive growth. From his analysis, Guinier concluded that the segregation zones have an irregular structure that gradually diverges from the matrix and tends to transform into a crystalline structure distinct from the initial precipitate. Subsequently, V. Gerold [77,78] proposed a model where the GP zone is composed purely of copper atoms between planes of pure aluminum which are closer to the origin than in the normal crystal. The displacement field weakens to zero over fourteen planes from the zone center. K. Toman [79] proposed an alternate model in 1957, suggesting that the copper atom concentration in various atomic planes of the GP zone decreased gradually, reaching 2.5 at.% at the tenth plane, with only a few planes displaced from their normal positions. However, in his 1960 publication [80], he acknowledged that his model requires an implausible number of copper atoms (more than 100% copper) to account for the observed diffuse scattering intensity. Thus, the Gerold models show significantly better agreement, especially when atomic plane displacements are increased.

Although the core structural models of GP zones are widely accepted, their precise exact structures and compositions continue to be subjects of research and discussion. The main point of contention lies in the exact Cu concentration and the displacement fields, which are influenced by the sensitivity of the characterization techniques and the interpretation of the data. This has led to a proliferation of models, each with its own merits and limitations.

One controversial issue is the actual strain field surrounding GP zones. Gerold’s proposed model for GP I zones [77,78] consists of a single layer of copper atoms on a (100) plane of the Al lattice, causing displacements of neighboring planes. In his model, the first matrix plane adjacent to the GP zone undergoes approximately 10% displacement, and the strain field gradually diminishes to zero by the 15th matrix plane. GP II zones (θ″ precipitates) are thought to consist of several layers of Cu atoms producing a similar strain field. In Toman’s model [79], the interplanar collapse vanishing at the fourth plane on either side of the zone, the displacements being roughly one-third those proposed by Gerold. The strain contrast from GP I and GP II zones has been observed by TEM by several researchers [81,82], with coherent strain fields rendered visible through diffraction contrast [83,84]. In the study of K. Doi [85], the first plane showed a displacement of approximately 0.15 Å toward the zone origin, while displacements in other planes were negligible. Their findings fall between those of Toman’s and Gerold: the zone structure is more two-dimensional than Toman’s model suggests, while Gerold’s model corresponds to a purely two-dimensional structure. In 1961, A.D. Thomas [86] observed 0.25 Å displacement exclusively in the first Al layer near the zone center, reconciling Gerold’s extended displacement field with Doi’s localized distortions. J. Parsons et al. [87] and V. Phillips [88] sought to determine lattice displacements along an axis through the center of GP I and GP II zones using the two-beam lattice fringe technique. Due to the nature of the strain field surrounding GP zones and its similarity to edge dislocation loops, a study was conducted to explore whether GP zones parameters could be determined using the weak-beam electron microscopy technique. A study by A. Fontaine et al. [89] using extended X-ray absorption fine structure (EXAFS) reported that the first plane adjacent to the GP I zone collapsed by 17% of the lattice parameter, possibly due to the vacancies. Although this experimental method has been questioned as less reliable [6], the same collapse amount was later found by X. Auvray et al. [90] using diffuse X-ray scattering, who observed that the facing Al planes collapse toward the GP I zone with the displacement falling more rapidly at successive layers than in the Gerold model. However, when M. Casanove-Lahana et al. [91] applied this value in HREM simulations of their experimental images, they obtained highly blurred results. The best fit was achieved with only 3.2% displacement. When measuring fringe displacement in images, Sato et al. [92] observed a 10% contraction in the center of the GP I zone, decreasing toward the zone edge. Matsubara and Cohen [93] also reported a value of 10%. Additionally, they discovered that the strain around the GP I site oscillated with distance from the zone and vanished near the 4th or 5th {100} atomic plane. These strain oscillations were also previously observed by V. Phillips [88] and T. Sato et al. [92] in electron microscope studies.

The Cu content within GP I zones and the adjacent matrix is another area of uncertainty. K. Doi [85] conducted a structural analysis of a GP zone in an Al-Cu alloy using Fourier transformation of the amplitude distribution around the reciprocal lattice point (200). For every atomic plane within the zone, this research quantified both the Cu atom concentration and the deviation from the matrix crystal’s net plane position. The analysis revealed that Cu content decreases after the second plane from the origin, indicating a quasi-two-dimensional structure for the zone. In 1961, A.D. Thomas [86] used Fourier analysis and Stieltjes planimetry to quantitatively reconstruct the electron density distribution of GP zones. His trial-and-error calculations revealed a three-layer structure with 75 at.% Cu in the central layer and 35 at.% Cu in adjacent layers, extending over an average diameter of 120 Å, an intermediate configuration between Gerold’s single-layer model and Doi’s quasi-2D structure. This model resolved previous discrepancies by showing that copper clustering occurs mainly within three atomic planes while maintaining coherent interfaces with the matrix, consistent with both X-ray diffraction intensities and hard-sphere atomic packing calculations. D. James and G. Liedl [94] analyzed GP I zones in an Al-1.67 at.% Cu alloy aged for different time, finding theta GP I zone diameters range from 35 to 63 Å, with Cu clustering confined to 3–5 planes. The central plane’s Cu content decreased from 57 at.% (6 h) to 30 at.% (36 h), while lattice plane displacements from the matrix extended beyond 8 planes from the center, with the maximum at the first plane. Auvray et al. [90], Matsubara and Cohen [93], and Gerold and Bubeck [6] reported 100% Cu in the GP I zones. In contrast, Fontaine et al. [89] proposed a model where the zone contains only 50% Cu. Hono et al. [95,96] conducted a detailed atom probe analysis on an Al-1.7 at.% Cu alloy aged at 130 °C for 16.7 h, finding that the Cu content in Cu-rich planes was even lower, ranging between 25 and 45%. In the depleted matrix (Cu solid solution), a Cu content of 0.75 at.% was identified, consistent with the 1 at.% measured by Fontaine et al. [89] using EXAFS in Al-2.0 at.% Cu and Al-2.5 at.% Cu alloys aged 90 °C for 50 h and 240 °C for 50 h. On the other hand, Auvray et al. [90] asserted that there is no significant Cu content in the matrix. The Cu content in GP I zones remains difficult to define, with ongoing controversy. Recently, M. Karlík et al. [97] reported via tomographic atom probe-field ion microscopy (TAP-FIM) that GP I zones with varying Cu concentrations (40–100%) coexist, consistent with observations previously reported. Of 12 GP I zones analyzed, 6 contained 100% Cu, 5 contained over 65% Cu, and 1 contained only 40% Cu.

It is well-documented that in Al-Cu alloy systems, GP zones precipitate from the supersaturated solid solution: during ageing, solute Cu atoms initially segregate on the (100) planes of the parent lattice and form plate-shaped GP zones [98]. However, direct observation of these zones remained challenging in the past. During the 1970s and 1980s, advancements in electron microscopy and other analytical techniques facilitated more in-depth investigations into θ-series precipitates. V. A. Phillips [88] employed a high-resolution electron microscope to investigate precipitation in an Al-3.0% Cu alloy aged at 130 °C, finding that GP zones seemed to be made up of copper-rich monolayers. He observed that initial monolayer GP I zones and parallel {100}-oriented clusters coarsen over time, forming isolated zones up to 300 Å in diameter with 9-atom-layer thickness and continuous electron diffraction streaks through {200} matrix spots along <100> directions. The study revealed a continuous transition from GP I to GP II to θ phases without abrupt microstructural changes, with θ′ particles nucleating and coarsening at the expense of GP zones, characterized by (001) lattice fringes at 5.8 Å and distinct (101)/(011) fringes at 3.3 Å forming lenticular shapes during coarsening, while GP II zones showed (001) fringes at 7.9 Å, and electron diffraction streaks from θ′ platelets along <001> through {110} matrix spots faded with prolonged aging, with brightfield/darkfield imaging resolving edge-on views of individual Cu-rich planes. In 1975, Victor A. Phillips utilized TEM to observe lattice fringes corresponding to c-planes within GP II zones, with a spacing of 7.9 Å. This resolution allowed distinction from the θ′ phase [99]. He observed that the fragmentation of continuous diffraction streaks, conventionally associated with GP II zones formation, occurred without detectable microstructural discontinuities. He attributed this absence of structural change to averaged diffraction effects from particle ensembles, demonstrating that the transition from GP I through GP II to the θ′ phase constitutes a continuous structural progression [99]. H. Yoshida et al. [100] used the weak-beam electron microscopy to investigate plate-like GP I and GP II zones in Al-Cu alloys. The findings from this study were compared with Gerold’s model of GP zones. In this work, the contrast of the zones was simulated using distortions from dislocation loops. But the Cu concentration within these loops was ignored, making the amount of Al plane collapse undetermined. N. Ajika et al. [101] used dynamical electron diffraction contrast calculations to interpret atomic-resolution images of GP I and II zones in Al-Cu alloys. The findings from this study directly validated Yoshida et al.’s copper monolayer model for GP I zones through experimental image matching. In this work, the contrast of GP II zones was simulated by introducing shear displacements into structural models. Through high-resolution transmission electron microscopy (HREM) [88,92], researchers have identified that GP zone variants consisting of two or more Cu atoms layers coexist with monolayer zones. This interpretation has further received support from computer analyzed diffuse X-ray scattering [90,93] and field ion microscopy (FIM) [95,102,103]. However, the volume fraction of these multilayer zones remain controversial, ranging from 13% [104] to 25% [90,93]. Conversely, in another FIM study, Wada et al. [105] examined numerous GP I zones but did not detect any single multilayer zone. However, during this period, the resolution of microscopes was only sufficient to study Al-Cu alloys under tilted illumination conditions. Regrettably, under such conditions, the intensity distribution on the bottom face of the crystal does not accurately reflect the atomic positions, making interpretation problematic [106]. Nevertheless, several studies were published, most notably those by Phillips [88], Yoshida et al. [106], Ajika et al. [101] and Sato et al. [92,107].

By the 1990s, electron microscopy and other analytical techniques had advanced further. Jouffrey and Dorignac [108] observed monolayer GP I zones using a 400 kV electron microscope under symmetrical illumination conditions. In simulations, they demonstrated that under varying defocus conditions, a monolayer zone may appear more complex than it actually is. In addition to the usual [l00] crystal direction, they also used a (110) zone axis orientation for the GP zone imaging, owing to the larger distance between atomic columns. An important point in their paper addresses the Cu content within the GP zones. In the (110) zone axis, they detected ordering in part of one GP I zone, leading to the conclusion that GP I zones exist with Cu concentrations below 100%. Jouffrey and Karlik [109] employed a 200 kV microscope equipped with a field emission gun to directly measure Cu content in GP I zones through characteristic X-rays excited by an extremely small electron probe (about 2 nm in diameter). The measurement procedure was based on a basic geometrical model. The issue of radiation damage during analysis was also addressed. Results indicated a Cu concentration of 50% or less in the studied GP I zones, provided the foil thickness was less than 20 nm. Employing the same model and accelerating voltage, Alfonso et al. [110] found only 25% Cu in GP I zones. Fujita and Lu [111] achieved new findings in GP zone studies by using a 300 kV microscope. Prior to the formation of GP I zones, three-dimensional clusters termed “GP pre-zones” composed of several Cu-rich planes separated by matrix planes were observed. Based on in situ irradiation-induced diffusion, these GP pre-zones can develop into GP I zones or directly transform into θ” phase. Regrettably, experimental images were not interpreted with simulations. In another study [112], they suggested a new mechanism for GP zones formation and their evolution to θ” phase. Estimation of Cu content in GP I zones and subsequent structures were approximately 50% in the most Cu-rich planes and 25% in adjacent planes. M. Takeda et al. [113] applied extended Hückel molecular orbital (EHMO) calculations to investigate the stability of GP I zones forming at low aging temperatures in Al-Cu alloys. To understand why such clusters preferentially form in {001} planes of the supersaturated Al matrix, they compared the stabilities of hypothetical clusters in (001), (110), and (111) crystallographic planes, based on quantum-mechanically calculated total energies of individual model clusters. The internal structure of GP I zones in Al-Cu alloys was also analyzed, and finally, the effects of additional elements on GP I zones stability were examined via EHMO calculations. M. Karlik and colleagues conducted a study on GP I zones in an Al-1.68Cu-1.10Mg-0.33Mn (at.%) alloy using high-resolution transmission electron microscopy (HRTEM). Most GP I zones were found to be composed of atomic monolayers, with disc diameters ranging from 4 to 10 nm. In the binary alloy, double-layer GP I zones and unstable disc-shaped particles on {111} planes were also identified [114].

Since it cannot directly observe Cu atoms by HRTEM, the interpretation of images made by modeling and simulation suggests the actual position of a precipitated layer(s). In the early 21st century, the high-angular annular dark-field scanning transmission electron microscopy (HAADF-STEM) method with sufficiently small probe size can eliminate ambiguities in identifying precipitates without losing resolution. T.J. Konno et al. [115,116] clearly resolved the intercalation of monatomic Cu plates in the early stage of GP zone formation, while in the so-called second stage (GP II), monatomic Cu layers sandwiching three Al layers were chemically identified. GP I zones can be double layered, although most are Cu mono-layers. GP II zones consisting of Cu monolayers separated by three Al layers were identified. Additionally, variants such as two Cu double layers separated by an A1 monolayer exist, indicating possible diversity in GP II zone structures. HAADF-STEM observations show that GP I zones are occasionally separated by three A1 layers, leading to the proposal that these pairs of GP I zones may represent an incipient stage of GP II zones. In recent years, the application of spherical aberration correctors has greatly facilitated the observation of the single copper atom layer in GP I zones using HAADF-STEM technology [97,117,118].

The longstanding debate over the precise structure and composition of GP zones stems from the intrinsic challenges of characterizing nanoscale, coherent features. Early discrepancies between models (e.g., Gerold vs. Toman) primarily arose from the indirect nature of X-ray diffraction data. Even modern techniques have their limitations: HAADF-STEM provides exquisite atomic column resolution but is less quantitative for composition, while APT can measure composition but may suffer from artifacts at interfaces. The reported coexistence of GP zones with a wide range of Cu concentrations (40–100%) suggests that the formation process may not lead to a single, well-defined structure but rather a spectrum of solute clusters. Resolving this conclusively likely requires a correlative approach, combining multiple atomic-scale techniques on the same specimen.

## 4. Recent Advances in θ-Series Precipitates Research

Recent advancements in electron microscopy and computational methodologies have significantly enhanced our atomic-scale understanding of θ-series precipitates, with notable progress manifested in several key aspects: the precipitation sequence, transformation mechanisms, interfacial characteristics of θ′/Al phases induced by alloying elements.

### 4.1. Precipitation Sequence and Transformation Mechanisms

Building on the historical context, recent advancements in atomic-scale characterization and computational methods have unveiled new insights into the precipitation sequence and transformation mechanisms of θ-series precipitates.

Regarding the development of the θ′ phase in regions populated by θ″ phases, existing research offers two distinct explanations. First, some studies propose that θ′ phase nucleates and grows independently, leading to the dissolution of the θ″ phase. Second, other studies suggest a continuous transformation mechanism, where the transition from θ″ to θ′ occurs through atomic reconfiguration within the precipitates [99,119]. Z. Shen et al. [120] explored the atomic-scale mechanism of the θ″ → θ′ phase transformation in Al-Cu alloys using aberration-corrected STEM. They clarified that in the presence of θ″ phases, θ′ forms via in situ transformation from θ″, retaining the same plate shape, size, and broad faces. The transformation initiates simultaneously at multiple sites within individual θ′’ precipitates through atomic rearrangement. During aging, the θ′ segments extend axially and coalesce into a full precipitate, while maintaining their plate shape, size, and interfaces. However, since θ′-Al_2_Cu has a higher Cu concentration than θ″-Al_3_Cu, some θ″ precipitates dissolve to supply the required Cu atoms. If adjacent θ′ segments coalesce with crystallographic disregistry, antiphase domain boundaries (APDBs) of the type a/2 <110> on {110}_θ′_ form within the final θ′ phase.

A greater number of studies have focused on the new intermediate phase structures formed during phase transformation processes within the precipitation sequence of θ-series precipitates.

S.K. Son et al. [121] proposed a modified precipitation sequence: supersaturated solid solution → quenched clusters → G.P. (I) → G.P. (II) → θʺ (independent of G.P. (II)) → θ′ → stable θ. Structural characterization revealed that quenched clusters and G.P. (I) are monolayer Cu-rich platelets, G.P. (II) is a bilayer structure, and θʺ consists of multiple (at least three) Cu layers.

Z. Q. Li et al. [122] identified the well-known θ″-Al_3_Cu phase (two Cu monolayers/GP zones separated by three {002} Al layers) and a previously unreported θ′′′-Al_6_Cu phase (two GP zones separated by six {002} Al layers), with the latter having a slightly lower number density than θ″-Al_3_Cu. Prolonged aging led to clusters of mixed θ″ and θ′′′ phases. First-principles calculations showed that the regular and irregular stacking of GP zones arises from enhanced Al-Al bonds induced by pre-existing GP zones, which promote new GP zones to form preferentially three or six {002} Al planes away. These enhanced bonds explain both current observations and previously reported precipitation phenomena related to GP zones, θ″, or θ′.

Y. F. Ouyang et al. [123] found that the formation enthalpies of all precipitates are negative, indicating thermodynamic stability. The size of GP I zones, predicted based on their formation enthalpy, is approximately 1.4 nm in diameter, consistent with experimental observations. Entropy plays a key role in stabilizing θ-Al_2_Cu relative to θ_C_′-Al_2_Cu. Regarding the influence of temperature and pressure, the formation free energies of θ″-Al_3_Cu, θ_C_′-Al_2_Cu, θ_D_′-Al_5_Cu_3_, and θt′-Al_11_Cu_7_ increase with temperature and pressure, while those of θ′-Al_2_Cu, θ_O_′-Al_2_Cu, and θ-Al_2_Cu decrease. Elastic constant calculations show that all considered precipitation phases are mechanically stable and anisotropic except θ_C_′-Al_2_Cu, with θ_D_′- Al_5_Cu_3_ having the highest Vickers hardness.

H. Liu [124] discovered that the θ′′-Al_3_Cu phase plays a crucial role in the precipitation process of Al-Cu alloys. This phase features a sandwich-like structure, with every two {200}_Cu_ layers spaced apart by three {200}_Al_ layers. The results reveal that this sandwich-like structure is energetically preferred due to competition between elastic strain energy and chemical bonding energy. To minimize the elastic strain energy of {200}_Al_ and {200}_Cu_ layers, {200}_Cu_ layers have a tendency to be spaced apart from one another.

L. Bourgeois et al. [8,125] identified intermediate structures involved in the α_Al_-to-θ′ transformation through atomic-scale imaging, proposing specific mechanisms such as the “zipperlike” process. This transformation mechanism enables interfacial migration through a complex zipperlike action of a series of individual atomic movements.

L. Gao et al. [126] identified two alternative atomic diffusion mechanisms based on the well-established two-step process (or shear process) [67,127]. One mechanism involves an interstitialcy diffusion process, where Cu atoms shuffle from base-centered to body-centered positions in the intermediate θ′_P1_ phase, accompanied by the diffusion of additional Al atoms occupying body-centered sites, reducing distortion and activation barriers. The other mechanism relies on direct diffusion of Al atoms, which migrate from existing layers to form a new atomic layer, with Cu columns remaining stable during the transformation from the intermediate θ′_T2_ phase to θ′. First-principles calculations confirm that these new diffusion processes are energetically favored. Both pathways differ significantly from the earlier theory, which posits direct Cu atom diffusion. Interface structure analysis demonstrates that Cu atoms can diffuse through a gap-filling mechanism, which is more direct and efficient compared to the two-step process. The newly discovered intermediate phase structure enables Al atoms to function as diffusion atoms, thus forming the θ′ phase.

An analysis conducted by Ma et al. [128] using atomic scale HAADF-STEM imaging and theoretical calculations suggests the existence of a novel precipitation pathway in pre-deformed Al-Cu alloys, specifically following the sequence Pre-θ′-2 → θ′. The strong structural similarities between Pre-θ′-2 and 1.5 c_θ′_-thick θ′ in terms of interfacial structure and thickness, combined with energetic calculations and preliminary in situ observations, lead the authors to propose a new precipitation pathway toward the key strengthening phase θ′.

Feng et al. [129] used atomic-resolution HAADF-STEM and first-principles calculations to characterize the aging evolution of θ′ phase in Al-Si-Cu-Mg alloys. Experimental observations reveal that the $\theta_{P}^{\prime }$ phase originates from the growth expansion of GP I zones. As aging time extends and atomic concentrations change, Cu atoms outside $\theta_{P}^{\prime }$ rearrange to form the $\theta_{P1}^{\prime }$ phase [130], followed by local formation of $\theta_{P2}^{\prime }$ precursor within $\theta_{P1}^{\prime }$ as Cu atoms diffuse inward. Notably, $\theta_{P2}^{\prime }$ can directly transform into θ′ phase without changing morphology, structure, or orientation relationships. Based on these findings, a novel aging precipitation sequence is proposed: SSSS → GP I → ${\theta_{P}^{\prime }\to \theta }_{P1}^{\prime }\to \theta_{P2}^{\prime }\to \theta^{\prime }$. The new precipitation sequence proposed in their work (SSSS $\to GPI\to \theta_{P}^{\prime }\to \theta_{P1}^{\prime }\to \theta_{P2}^{\prime }\to \theta^{\prime }$) differs significantly from the currently widely accepted precipitation transformation process: SSSS → GP I → GP II $\to \theta^{\prime \prime }\to \theta^{\prime }$.

I. Zuiko and R. Kaibyshev [131] investigated how tensile strain (1–7%) affects the decomposition sequence and mechanical properties of the AA2519 alloy. Pre-straining hinders GP zone and the θ″ phase formation and strongly promotes θ′ phase precipitation on dislocations. As pre-strain increases, the nucleation mechanisms of θ′ and Ω phase from homogeneous to heterogeneous nucleation on dislocations and θ′ phase/Al matrix interfaces, respectively. Agglomerates consisting of platelets with {100}_Al_ and {111}_Al_ habit planes nucleate and grow simultaneously under peak-aging. After pre-strains ≥  3%, the following precipitation sequences occur: SSSS → GP zones → θ″ phase → θ′ phase + Ω_II_ phase → θ′ phase → θ phase, and SSSS → {111} clusters → Ω_I_ phase → θ phase

L. Zhou et al. [130] demonstrated that during aging at elevated temperatures (≥200 °C), θ′-phase precipitation in Al-Cu alloys follows a distinct pathway independent of classical precursors (GP zones, θ″). This high-temperature sequence evolves as: SSSS → θ′_HTP1_ (Al_5_Cu) → θ′_HTP2_ (Al_4_Cu_2_) → θ′ (Al_2_Cu).

J. Yan et al. [132] systematically investigated the precipitation behaviors and mechanical properties of Al-Cu-(Sc) alloys. A secondary high-temperature precipitation scenario of the θ′ phase exists in undeformed bulk Al-Cu-(Sc) alloys during aging at elevated temperatures (≥250 °C), following the sequence: SSSS → θ′_S-HTP_ → θ′. The plate-like transitional θ′_S-HTP_ precipitate, composed of three Cu atomic layers sandwiched by two Al atomic layers, is one of the three distinct precursors of the θ′ phase identified to date. The θ′_S-HTP_ precipitate can genetically evolve into a θ′ precipitate during thermal aging without changing its morphology or orientation.

The discovery of these alternative precipitation pathways fundamentally challenges the classical, linear sequence. It is now evident that the dominant pathway is not universal but is dictated by a complex interplay of thermodynamic driving forces and kinetic pathways, which are sensitive to alloy composition and processing history. A critical unanswered question is whether these pathways are mutually exclusive or can operate concurrently within a single alloy, contributing to a heterogeneous microstructure. Understanding the thermodynamic and kinetic factors that select for one pathway over another is crucial for the targeted microstructural design.

### 4.2. θ′/Al Interface

#### 4.2.1. θ′/Al Interface Characteristics

Beyond the precipitation pathways themselves, the interfacial characteristics between θ′ and the Al matrix have emerged as key factors controlling precipitate stability and coarsening resistance.

Given the critical role of θ′/Al interfacial stability in governing the growth and coarsening of strengthening precipitates in Al-Cu alloys, researchers have focused on characterizing these interfaces. V. Vaithyanathan et al. [133] developed a multiscale model integrating first-principles calculations, cluster expansion, Monte Carlo simulations, and phase-field modeling to study θ′ precipitate growth and coarsening. First-principles calculations provided critical energetic parameters: coherent and semi-coherent interfacial energies (190 and 600 mJ/m^2^ via LDA), stress-free misfit strains (−0.57% for coherent faces, −5.1% for 2cθ′:3aAl semi-coherent rims), and elastic constants. Phase-field simulations demonstrated that equilibrium θ′ morphology (high-aspect-ratio plates) is governed by both interfacial energy anisotropy (3:1 semi-coherent/coherent) and elastic energy anisotropy, rather than interfacial effects alone. Quantitative predictions of precipitate length, thickness, and aspect ratio evolution showed good agreement with experimental data. Hu et al. [134] calculated θ′/Al interfacial energies and critical nucleus characteristics using modified embedded-atom method (MEAM) potentials. Coherent interfacial energy was determined as 0.156 J/m^2^, while semi-coherent energy for 3:2 θ′:Al unit cell matching was 0.694 J/m^2^, consistent with first-principles results. Both theoretical nucleation analysis and atomistic simulations identified a plate-shaped nucleus with a semi-coherent 3:2 matching interface (where three FCC Al unit cells align with two θʹ unit cells along the plate edge) as energetically favored. This configuration exhibited a critical radius of ~2.4 nm and a nucleation barrier of ~2.3 × 10^−18^ J. GP zones were confirmed as precursors with lower nucleation barriers (<0.8 nm critical size).

Laure Bourgeois et al. [125] revealed that the coherent interface of the θ′ (Al_2_Cu) precipitate in Al-Cu alloys has a structure different from previous assumptions. Early-stage precipitates exhibit a GP (I)-zone-like interface with Cu atoms occupying interstitial sites at (0.5,0.5,0) in the θ′ unit cell, while late-stage interfaces are less Cu-enriched and approach the bulk θ′ structure. Density functional theory (DFT) calculations confirm that interfacial Cu segregation is energetically favorable but less so than solute incorporation via θ′ thickening. These findings indicate that the GP I zone-like interface serves as a structural intermediate state during θ′ phase thickening, challenging conventional models of precipitate growth kinetics in Al alloys. K. Kim et al. [135] explored the interfacial stability of θ′/Al in Al-Cu alloys, emphasizing that designing low-energy interface structures is critical for studying θ′ precipitate growth and coarsening. They examined the energetics of the coherent (001)_θ′_//(001)_Al_ interface and semi-coherent (010)_θ′_//(010)_Al_ interface, revealing that interstitial Cu atom occupancy at the coherent interface increases interfacial energy likely driven by kinetic effects. For the semi-coherent interface, interfacial energy was found to be relatively independent of interfacial configurations and misfit strains up to 10-unit cells of Al. M. F. Chisholm et al. [136] found that the {110} semi-coherent interfaces contain an array of misfit dislocations arranged into two structural units, which accommodate nearly all misfit between the Al matrix and θ′ phase. These units are characterized by 2 c_Al_ unit vectors matching 1.5 c_θ′_ unit vectors, and 3 c_Al_ unit vectors matching 2 c_θ′_ unit vectors. The residual misfit in the planar, periodic {110} semi-coherent Al/θ′ interface is only 0.25%. Additionally, excess Cu from both the Al matrix and θ′ precipitates segregates to the compressed edge of dislocation cores at three specific interface sites, and the semi-coherent interface structure plays a key role in stabilizing of metastable strengthening precipitates in critical structural materials.

In addition, Al/θ′ phase interfaces also exert an influence on other precipitated phases. In the study of I. Zuiko and R. Kaibyshev [131], the Ω_I_-phase the AA2519 alloy nucleates homogeneously with an aspect ratio (AR) of ~120. The Ω_II_-phase nucleates on Al/θ′ phase interfaces with an AR of ~ 35. Dissolution of the Ω_II_-phase leads to overaging.

#### 4.2.2. θ′/ Al Interface Regulation by Alloying Elements

Beyond intrinsic interface characteristics, alloying element segregation of at these interfaces emerges as a key mechanism for modulating diffusion kinetics and enhancing θ′ phase stability. Segregation of alloying elements at the Al/θ′ interface modulates Cu atom diffusion kinetics, thereby affecting the thermal stability of the θ′ phase.

In their 1974 study on Al-Cu alloys [137], Sankaran and Laird explored trace Cd, In, and Sn effects on θ′ plate precipitates: these elements primarily segregated to θ′ edges, reducing θ′ edge interfacial energy by a factor of 7 (Cd), 9 (In), and 15 (Sn). In Al-Cu-Cd/In, θ′ ledge density was higher than in pure Al-Cu due to more precipitate impingements from higher precipitate density. Kinetically, the elements did not affect θ′ thickening and had no early-stage impact on θ′ lengthening. D. Shin et al. [138] investigated solute segregation at the Al/θ′-Al_2_Cu interface in Al-Cu alloys using an extensive first-principles database of segregation energies for 34 elements. They found that segregation energies are strongly correlated with solute atom size, volume, and solubility in θ′. Elements with high solubility in both Al and Cu sublattices of θ′ (e.g., Mn, Fe, Co) due to ideal mixing show favorable segregation at semi-coherent interfaces across platelets. L1_2_-trialuminide formers (Ti, Zr, Hf, Sc) exhibit ideal mixing only in the Cu sublattice and moderate positive mixing in Al sublattices; they are generally repelled at interface platelets (Ali) but strongly segregate at outer platelets (Ali-1). Elements with strong affinity for vacancies in Al (Cd, In, Sn, Sb) strongly segregate at both interfaces, potentially suppressing θ′ coarsening and enhancing interfacial bonding. Elements improving θ′ high-temperature stability (Mn, Zr, Sc) segregate more favorably at semi-coherent than coherent interfaces. Mn (miscible in both θ′ sublattices) and Zr (soluble only in Cu sublattices) together extend temperature limits; Fe and Co may act similarly to Mn. L1_2_ formers (Zr, Hf, Sc) can synergize with 3d transition metals (Mn, Fe, Co) to further stabilize θ′. Their results are limited to theoretical calculations without corresponding experiments. However, many other researchers have conducted theoretical calculations and related experiments on microalloying.

Among microalloying elements, Sc has been extensively studied, with numerous experimental and theoretical works demonstrating its potent effects on θ′ precipitation and stability. B.A. Chen et al. [139] studied an Al-2.5 wt% Cu alloy containing 0.3 wt% Sc, finding that Sc solute atoms significantly enhanced the uniform precipitation of fine θ′ particles, resulting in an approximately 90% increase in peak aging hardness compared to the Sc-free alloy. L. Jiang et al. [140] found that minor Sc addition exerts a microalloying effect on Al-Cu alloys, but this effect is strongly length-scale dependent: the smaller the grain size, the more active the microalloying effect, promoting intragranular precipitation and reducing intergranular precipitation. In Al-Cu alloys, as the grain length scale decreased, intergranular θ precipitation gradually dominates at the expense of intragranular θ′ precipitation. Compared to Sc-free counterparts, the yield strength of post-aged CG, FG, and UFG Al-Cu-Sc alloys increased by ~36 MPa, ~56 MPa, and ~150 MPa, respectively, while tensile elongation increased by ~20%, ~30%, and 280%. C. Yang et al. [141] also investigated the influence of Sc solute partitioning on the microalloying effect and mechanical properties. In their study, atom probe tomography (APT) revealed Sc segregation at the θ′/matrix interface, which exerts a significant microalloying effect by promoting the precipitation of the θ′-Al_2_Cu strengthening phase during ageing. The Al-2.5 wt% Cu-Sc alloy exhibits a room-temperature yield strength approximately 2.2 times that of the Sc-free alloy and 1.8 times that of the Al-1.5 wt% Cu-Sc alloy, with improved high-temperature mechanical properties attributed to enhanced θ′ coarsening resistance induced by Sc segregation. Y. H. Gao et al. [142] explored creep resistance in Sc-microalloyed Al-Cu alloys, finding that strong Sc segregation at the θ′/matrix interface significantly stabilizes θ′ even during creep at 300 °C, resulting in extremely high creep resistance. They [143] also found the Sc-rich entities preferentially form in regions adjacent to θ′ precipitates, contributing to the refinement of θ′ precipitation in the Al-Cu-Sc alloy. At higher aging temperatures (~300 °C), the pre-formed Sc-rich entities are absorbed by adjacent θ′ precipitates. Furthermore, they [144] explored the assembly of dual precipitates (θ′ and Al_3_Sc) in multi-microalloyed Al-Cu alloys to improve high-temperature resistance. θ′ stabilization in Sc-Zr or Sc-Si alloys relates to modified multiple solute segregation at the θ′/Al interface, reducing interfacial free energy. Results showed that Sc-Si microalloying retards θ′ coarsening but accelerates Al_3_Sc growth, leading to limited improvement in high-temperature resistance. In contrast, Sc-Zr microalloying simultaneously stabilizes both θ′ and Al_3_Sc precipitates, markedly enhancing softening resistance and outperforming Sc or Sc-Si strategies. C. Yang et al. [145] investigated the effect of Sc microalloying on Al-2.5 wt% Cu alloys with varying Sc additions (0, 0.1, 0.3, 0.5 wt%). Results showed that Sc addition effectively promotes θ′ precipitation, reducing its size and narrowing the size distribution. Enhanced creep resistance in the 0.5 wt% Sc alloy stems from a nanostructural Sc-based hierarchy, comprising Al_3_Sc dispersoid/heterogeneous θ′ precipitate units, homogeneous θ′ precipitates, strong Sc segregation at θ′/matrix interfaces, and Sc clusters. D. Zhang et al. [146] conducted first-principles studies on Sc-doped θ′/Al interfaces in Al-Cu alloys. They modeled Sc-doped semi-coherent and coherent θ′/Al interfaces, with Sc doped in the Al slab (S1 site) and θ′ slab (S2 site). Analysis showed that Sc doping at the S1 site significantly reduces interface energy and increases work of adhesion, especially the coherent interface with Sc at the S1 site (occupied by interstitial Cu atoms), which has excellent bonding strength. Electronic structure analysis revealed strong Al-Cu bonds at interfaces with Sc at the S1 site, while Al-Al bonds form when Sc is at the S2 site; these strong bonds are key to enhancing the strength of doped interfaces. In the work of J. Yan et al. [132], Sc atoms tend to segregate to the next nearest neighbors of the coherent interfaces between Al and θ′, as well as between Al and θ′_S-HTP_. Sc addition significantly increases the survival rate of θ′_S-HTP_ precipitates while suppressing the growth of much thicker primary θ′_HTP_ precipitates. Consequently, Sc microalloying refines θ′ precipitates and enhances the thermal stability of the alloy. Y.H. Gao et al. [147] comprehensively studied the individual effect of Sc and the combined effect of Sc and Fe on microstructural evolution during homogenization and the resulting mechanical responses. Results showed that a single minor Sc addition refines as-cast Al-Cu alloy grains and induces Sc enrichment in micro-sized Al/θ eutectics. Co-addition of minor Fe and Sc resulted in grain sizes and micro Sc-segregation similar to single Sc addition. Y. H. Gao et al. [148] explored solute repositioning to tune multiple microalloying effects in an Al-Cu alloy with minor Sc, Fe, and Si additions. They found that multiple Sc-Fe-Si segregation at the θ′/matrix interface preferentially forms in the as-aged alloy. During subsequent high-temperature creep, this interfacial segregation is rapidly reinforced by solute repositioning, with solutes diffusing from both inner θ′ precipitates and outer matrices to accumulate at the interface, limiting interfacial migration. It is worth noting that references [139,141,142,143,144,145,147,148] all mention that the balance between Sc solutes (aiding θ′) and Al_3_Sc (competing for Sc) affects the alloy’s precipitation behavior and mechanical properties.

In parallel to Sc, Si is another significant microalloying element, influencing θ′ behavior through mechanisms such as catalyzing nucleation and reducing interfacial energy. X. Gao et al. [149] found that adding Si to the Al-4Cu-0.3Mg alloy significantly increases its maximum hardness; the peak hardness of Al-Cu-Mg-Si alloys was correlates with the coexistence of uniformly distributed, fine-scale {100} θ′ plates and <100> α laths of the Q phase (Al_4_Cu_2_Mg_8_Si_7_). Moreover, incorporating 0.2 wt% Si into the Al-4Cu-0.3Mg-0.4Ag alloy completely inhibits the formation of the θ phase, which dominates in the quaternary alloy. D. Mitlin et al. [150] studied the effect of Si on θ′ precipitation in Al-2Cu-1Si alloys. During aging at 225 °C, Si precipitates first and catalyzes θ′ nucleation, leading to smaller, denser θ′ particles with lower aspect ratios and slower coarsening than in binary Al-2Cu alloys. The Al/θ′ interfacial tension was estimated at 85–96 mJ/m^2^ using the KH theory. Elastic interactions between Si and θ′ suppress coarsening, while Cu in the ternary alloy promotes Si precipitate twinning through a “step-poisoning” effect. A. Biswas et al. [151] investigated the compositional evolution of GP II zones and θ′ (Al_2_Cu) precipitates, as well as solute segregation at Al/θ′ interfaces in Al-1.7 at.% Cu alloys with ~200 at. ppm Si. Si partitions to both GP II zones and θ′ precipitates, with significant segregation at the Al/θ′ interface at 533 K, transitioning from non-equilibrium delocalized to equilibrium localized profiles with increasing temperature. First-principles calculations confirmed strong thermodynamic driving force for Si to occupy Cu sites in θ′, reducing interfacial free energy and enhancing nucleation kinetics. L. Liu et al. [152] systematically investigated the θ′ nucleation and growth in the Si-containing alloy during isothermal aging at 180 °C. The formation of fine Q″-type precipitates serves as heterogeneous nucleation sites for θ′ precursors, accelerating aging kinetics and enhancing early-stage strength. The small size of Q″-type precipitates restricts θ′ thickening, leading to a large proportion of θ′ precipitates with a specific thickness of 2cθ′ that remains stable during aging. L. Jiang et al. [153] found that when Si segregates at the A1 site (Al slab) of the semi-coherent interface system, the segregation energy is the most negative, significantly reducing the total system energy and enhancing the interfacial bonding force. Electronic structure analysis showed that when Si occupies the A1 site, Al-Cu and Al-Al bonds form in the system, and the formation of these strong bonds is crucial for improving interfacial bonding strength. Results indicate that Si can act as a synergistic element to reduce the θ′/Al interfacial energy, thereby lowering the coarsening driving force of the θ′ precipitated phase.

Cd, long recognized for its coarsening inhibition effect, operates primarily through vacancy trapping and interfacial segregation, offering another pathway for θ′ stabilization. As early as 1971, J. D. Boyd and R. B. Nicholson [32] studied the coarsening behavior of θ″ and θ′ precipitates in two Al-Cu alloys (Al-4% Cu and Al-4% Cu with trace Cd addition) using transmission electron microscopy. Results showed that θ″ coarsening behaviour was in qualitative and quantitative agreement with theory. In contrast, θ′ coarsening behavior was anomalous: initially, its coarsening rate was much faster than predicted (suggesting short-circuit diffusion), but it eventually decreased sharply to an almost negligible value. Trace Cd addition reduced the coarsening rate of θ′ by a factor of 5. Yisen Hu et al. [154] found that Cd in Al-Cu alloys can trap vacancies, extending their influence on Cu diffusion in the Al matrix, and tends to segregate at θ′/Al interfaces, reducing interfacial energy and promoting θ′ nucleation. The model describes the precipitation process as involving five stages: incubation, nucleation, growth, Cd release, and coarsening. Y. Hu et al. [155] investigated θ′ (Al_2_Cu) precipitation behavior in Al-Cu-Cd alloys using phase-field simulations, revealing that Cu diffusion in the Al matrix is dominated by Cu-Cd-vacancy clusters due to their unique formation effects. Cd atoms segregate at the Al/θ′ interface, reducing the interfacial energy of the θ′ phase. The study compared multiple combinations of coherent and semi-coherent interfacial energies to analyze θ′ growth kinetics. Results showed that the diameter-to-thickness ratio of θ′ primarily depends on coherent interfacial energy, and the equilibrium diameter increases monotonically with this ratio. H. W. Bai et al. [156] explored the application of trace Cd addition (∼0.2 wt%) in Al-Cu alloys to enhance mechanical properties by introducing hybrid θ″ + θ′ precipitates, with the outcome being regulated by solid-solution temperature. At low solid-solution temperature (T = 500 °C), Cd microalloying only results in the formation of undesirable dual precipitates of θ″ and θ′, which have similar sizes and homogeneous distribution. Raising the temperature to 530 °C leads to the generation of dense Cd-rich nanoparticles. These nanoparticles significantly facilitate the formation of hybrid θ″ + θ′ precipitate with a bimodal distribution by providing numerous heterogeneous Cd-rich nucleation sites. In the Al-Cu-Cd alloy treated at 530 °C, Cd elements are concentrated at the periphery of both θ″ and θ′ particles.

Synergistic microalloying, particularly with Mn and Zr, has proven highly effective for stabilizing θ′ precipitates at elevated temperatures. D. Tsivoulas and J.D. Robson [157] studied on the heterogeneous Zr solute segregation in Al-Cu-Li alloys. Notably, Zr did not interact with the θ′ phase, but it interacted with the equilibrium θ phase, generating two types of particles: one containing only Zr and another containing both Zr and Mn. G. Niu et al. [158] observed an inclined θ′ morphology with terraced Cu/Mn-rich multilayers in Al-Cu based alloys. These inclined θ′ precipitates have an angle of ~3.1° and lattice parameters a = 0.385 nm and c = 0.535 nm, with Mn significantly enriched at the terraced coherent interfaces. This helps maintain the (001)_θ′_//(001)_Al_ coherent relationship despite large misfit strains. The interfacial energy between the coherent Cu/Mn-rich (001)_θ′_//(001)_Al_ interface and semi-coherent (010)_θ′_//(010)_Al_ interface is closer than that of the normal (001)_θ′_//(001)_Al_ interface, weakening the anisotropic interfacial energy of θ′ with Cu/Mn-rich layers. A. Shyam et al. [159] investigated the microstructural stability of cast AlCuMnZr alloys at elevated temperatures. Atomic-scale characterization and first-principles calculations demonstrate that microalloying with Mn and Zr is critical for stabilizing high-energy interfaces. The key mechanism involves Mn and Zr segregation toθ′ precipitate-matrix interfaces, inhibiting coarsening and transformation and allowing metastable precipitates to remain stable at higher homologous temperatures. Similarly, J. D. Poplawsky et al. [160] found that an Al-Cu alloy with combined Mn and Zr additions can withstand prolonged exposure up to 350 °C, whereas adding Zr or Mn alone only improves stability to 200 °C and 300 °C, respectively. They revealed that Mn additions stabilize θ′ precipitates long enough for slower-diffusing Zr atoms to segregate to coherent θ′ interfaces.

To systematically understand the distinct segregation behaviors and strengthening effects of different alloying elements, comparative first-principles studies have been conducted. X. Chen et al. [161] investigated the segregation origins of Cd, Si, Sc, Zr and their effect on Al/θ′ interface strength in Al-Cu alloys via first-principles calculations. Chemical contribution dominates their oscillatory segregation on the Al matrix side, while both chemical and mechanical contributions govern that on the θ′ side. Segregation tendencies vary with elements and interfaces, related to charge accumulation with host atoms: Sc, Zr show strong segregation on the Al side; Si favors θ′ side; Cd has weak tendency at (001) interface but strong at (010) interface. Segregation tendencies of Si, Sc, Zr enhance with coverage, while Cd’s decreases. Strengthening is mainly chemically driven: Sc, Zr increase interface strength via strong electronic interactions; Cd weakens it due to weak interactions. The (001) interface with Sc or Zr has better ductility than with Cd or Si, providing a strategy to improve Al-Cu alloys’ mechanical properties. M.V. Petrik et al. [162] conducted a systematic investigation into the interactions between alloying elements (Si, Mg, Mn, Zr, Zn), vacancies, and the coherent interfaces of the θ′ phase in Al-based alloys using ab initio supercell calculations. Their calculations revealed that the interface structure with a half-filled interfacial Cu layer exhibits lower energy (0.1 eV per structural vacancy) compared to that with a fully filled Cu layer, and the extent of interface reconstruction is dependent on the presence of vacancies. Vacancies within the interfacial Cu layer play a crucial role in the interaction between solutes and the coherent θ′ phase interfaces. Solute-interface interaction energies are significantly weaker for elements with closed (Cu, Zn) or empty (Mg, Si) d-electron shells compared to d-transition metals (Mn, Zr).

J. Wang et al. [163] conducted a first-principles study on the influence of multi-element (Ca, Co, Si) doping on the mechanical properties of the θ (Al_2_Cu)/Al interface in Al-Cu alloys. They found that the Al2-terminated interface is the most stable in the Al-Al_2_Cu configuration. Compared to the pristine interface, doping with Ca, Co, or Si enhances the elongation of the interface structure, which is attributed to electron enrichment around the dopant and Al atoms. Among them, Co doping also improves the yield strength of the interface, and the Co-doped interface exhibits the largest fracture toughness, indicating the highest ductility. Generalized stacking fault energy (GSFE) calculations for shear resistance estimation showed that slip is more difficult at the Ca-doped interface than at others.

Additionally, research on some alloying elements remains relatively unexplored.

L. Bourgeois et al. [164] studied the structure and thickness of θ′ precipitates in an Al-1.7 at.% Cu-0.01 at.% Sn alloy to explore how Sn promotes θ′ nucleation. HAADF-STEM imaging showed that θ′ platelets newly nucleated at 160 and 200 °C exhibit discrete “magic” thicknesses, which correspond to minima in residual volumetric and shape misfit strain. This phenomenon is unique to Sn-assisted nucleation. Direct evidence indicated that Sn does not accommodate volumetric misfit strain but can reduce either the interfacial energy of θ′ or the transformation shape strain associated with intermediate thicknesses, thereby promoting the nucleation of θ′ with specific magic thicknesses. In the study of R.H. Wang et al. [165], it showed that Sn addition promoted the dispersion of θ′-Al_2_Cu precipitates, leading to a refined size and increased number density. APT examinations revealed that the Sn microalloying mechanism primarily involves the heterogeneous nucleation of θ′ precipitates on Sn particles.

R. Yoshimura et al. [166,167] found the structure and precipitation behavior of θ′ phase in aged Al-Cu alloys are strongly dependent on Li content. In the 1.6 wt–Li alloy, θ′ precipitates retain the lattice parameters of the Al-Cu binary system, while the 2.4 wt%-Li alloy exhibits atypical θ′-related structures. GP-I zones are the dominant early precipitates in both alloys during aging, stabilized by flanking lenticular δ′ particles in an anti-phase relationship, even at elevated temperatures. GP-II zones are rare in both alloys. SY Duan et al. [168] explored composite precipitates in low Li-content Al-Cu-Li alloys, where δ′-precipitates envelop GP zones and θ′-precipitates. They found these composites share Cu-Li bonded interfaces with Li atoms as the second nearest neighbors to Cu atoms. Two relationships—”anti-phase” and “in-phase”—exist between sideward δ′-precipitates in δ′/θ′/δ′ structures, determined by the number of Cu layers in inner θ′-precipitates; these relationships switch when θ′ thickens from even to odd (or vice versa) via layer-by-layer growth, involving structural modifications until δ′ adapts to optimized interfaces. This behavior suppresses thermal coarsening, maintaining fine microstructures. First-principles calculations showed composite formation lowers system energy significantly by reducing interfacial energy through replacing θ′/Al interfaces with Al/δ′/θ′/δ′/Al interfaces. In the study of Z. Liu et al. [169], highly diffusive excess Li was found to segregate to the early-precipitated β′(L1_2_-Al_3_Zr) nano-phase, forming a tentative δ′(L1_2_-Al_3_Li) shell in the early aging stage. This δ′ shell promotes GP-zone precipitation along (100) facets but is metastable and eventually dissolves into β′ over time. As a result, GP-zones can independently evolve into θ″ and θ′ during prolonged aging. Additionally, excess Li weakly segregates to θ′ interfaces, forming an “in-phase” or “anti-phase” δ′ shell. J. Ma et al. [170] systematically evaluated the effect of the Cu/Li ratio (ranging from 0.5 to 1.4) on aging hardening and precipitation behavior in Al-Cu-Li alloys containing specific rare earth elements (Sc, Zr, Ce). A new Li-rich phase, termed the GP-Li zone, with a hexagonal morphology was identified in alloys with Cu/Li ratios of 0.5 and 1. High-density GP-Li zones are fully coherent with the Al matrix, uncovering a novel precipitation path for Li atoms in supersaturated solid solutions. Additionally, the GP-Li zone promotes the nucleation of θ′ phases at interfaces, suggesting an alternative nucleation mechanism.

Y. Chen et al. [171] used atomic-resolution Z-contrast STEM and first-principles calculations to investigate enhanced θ′ precipitation in Al-Cu alloys with trace Au additions. The findings from this study demonstrated accelerated aging kinetics through Au-induced microstructural modifications. In this work, the strengthening mechanism was attributed to reduction in θ′ critical nucleation dimensions enabling single-unit-cell precipitates, lowered nucleation barriers promoting homogeneous θ′ distribution, and preferential Au substitution at Cu sites within θ′. Y. Zheng et al. [172] discovered a previously unreported sandwich structure formed in aged Al-Cu and Al-Cu-Au alloys. This sandwich structure comprises a stack of regularly spaced plates of metastable precipitate phases: GP zones, θ″, and θ′. Within the structure, the separation between the broad surface of θ′ and its adjacent GP zone, as well as between two neighboring GP zones, is consistently three {002}_α_ planes. The structure is observed across θ′ precipitates of varying thicknesses. Based on experimental results and calculations, the formation of this sandwich structure is attributed to Cu atom segregation at the θ′/Al interface, rather than the misfit associated with θ′ formation. In the ternary Al-Cu-Au alloy, Au atoms are primarily distributed in the central part of the θ′ plate, not at the θ′/Al interface or within the GP zone. Calculations validate this observation and further reveal that Au atoms energetically favor substituting Cu (rather than Al) atoms within θ′.

J.M. Rosalie and L. Bourgeois [173] used HAADF-STEM to study θ′ (Al_2_Cu) precipitates in Al-Cu-Ag alloys, finding that they nucleate on Ag-enriched dislocation loops with γ′ (AlAg_2_) assemblies. The Al-θ′ interface has a 2-atomic-layer Ag segregation layer that lower interfacial energy, and θ′ lateral growth depletes Ag from γ′ precipitates, degrading γ′ assemblies.

Q. Cai et al. [174] fabricated eutectic alloys from the quaternary Al-Cu-Si-Ni system and found that Ni (∼1.7 at%) dissolves into tetragonal θ-Al_2_Cu. DFT calculations showed that configurational entropy stabilizes this level of randomly substituted Ni at Cu sites in the θ-Al_2_Cu lattice at high temperatures. Post-solidification annealing revealed that the ternary eutectic microstructure has better thermal stability than the corresponding Al33Cu (wt%) binary eutectic. Ni solution in θ-Al_2_(CuNi) contributes to this stability.

A comparative analysis of microalloying elements reveals distinct stabilization mechanisms: Sc and Si operate primarily by thermodynamic stabilization through interfacial energy reduction; Cd exerts a kinetic control by trapping vacancies and slowing coarsening; and the Mn/Zr combination demonstrates a potent synergistic effect for high-temperature stability. However, a significant challenge in multi-component alloy design is managing elemental interactions. For instance, while Sc is highly beneficial, excess Sc can lead to the formation of Al_3_Sc precipitates that compete with θ′ for solute atoms. Future research must move beyond studying single-element effects and focus on the complex interplay and potential antagonism between multiple microalloying elements to establish predictive design rules.

## 5. Summary and Outlook

This review has traversed the historical developments, fundamental mechanisms, and recent advances in θ-series precipitate research, highlighting both established knowledge and ongoing controversies. Early studies laid the groundwork by identifying age hardening in Duralumin and establishing the precipitation sequence, while modern techniques such as high-resolution electron microscopy and computational methods have deepened understanding of atomic-scale structures, phase transitions (e.g., θ″ → θ′), and interfacial characteristics of θ′/Al. Key findings include the clarification of precipitation pathways (e.g., new sequences in Al-Si-Cu-Mg and high-temperature-aged Al-Cu alloys), the role of alloying elements (Sc, Si, Cd, Mn, Zr) in regulating θ′ stability via interfacial segregation and vacancy interactions, and ongoing controversies such as the precise structure of GP zones and mechanisms of multi-element interactions.

Despite these advances, several challenges remain unresolved, pointing to promising avenues for future research. Future research should focus on addressing critical challenges to advance alloy design. First, developing comprehensive models for θ′ coarsening kinetics, integrating interfacial energy, diffusion barriers, and alloying effects, is essential for improving high-temperature stability. Second, elucidating synergistic mechanisms of multi-element microalloying through in situ characterization and multi-scale simulations will accelerate the development of high-performance alloys. Additionally, engineering θ′/Al interfaces via controlled solute segregation and resolving remaining ambiguities in GP zone structures and phase transitions using advanced microscopy and modeling will further unlock the potential of θ-series precipitates, enabling stronger, more stable aluminum alloys for aerospace, automotive, and structural applications.

## Figures and Tables

**Table 1:** The crystallographic parameters of GP zone, θ″, θ′, and θ.

Phase	Composition	Crystal Structure	Lattice Parameters (nm)	Orientation Relationship	Coherency
GP Zone	Cu-rich layer	Disc on {100}_Al_	a ≈ 0.405	Fully coherent with matrix	Coherent
θ″	Al_3_Cu	Tetragonal	a = 0.404, c = 0.768–0.780	(001)_θ″_//(001)_Al_, [100]_θ″_//[100]_Al_	Coherent
θ′	Al_2_Cu	Body-centered tetragonal	a = 0.404, c = 0.580	(001)_θ′_//(001)_Al_, [100]_θ′_//[100]_Al_	Semi-coherent
θ	Al_2_Cu	Body-centered tetragonal	a = 0.6066, c = 0.4874	(001)_θ_//(001)_Al_, [100]_θ_//[100]_Al_	Incoherent

## Data Availability

No new data were created or analyzed in this study. Data sharing is not applicable to this article.
